# Heritability of Subcortical Volumetric Traits in Mesial Temporal Lobe Epilepsy

**DOI:** 10.1371/journal.pone.0061880

**Published:** 2013-04-23

**Authors:** Saud Alhusaini, Cathy Scanlon, Lisa Ronan, Sinead Maguire, James F. Meaney, Andrew J. Fagan, Gerard Boyle, Gabor Borgulya, Parameswaran M. Iyer, Paul Brennan, Daniel Costello, Elijah Chaila, Mary Fitzsimons, Colin P. Doherty, Norman Delanty, Gianpiero L. Cavalleri

**Affiliations:** 1 Molecular and Cellular Therapeutics Department, Royal College of Surgeons in Ireland, Dublin, Ireland; 2 Brain Morphometry Laboratory, Epilepsy Programme, Beaumont Hospital, Dublin, Ireland; 3 Clinical Neuroimaging Laboratory, Department of Psychiatry, National University of Ireland, Galway, Ireland; 4 Brain Mapping Unit, Department of Psychiatry, University of Cambridge, Cambridge, United Kingdom; 5 Radiology Department, Beaumont Hospital, Dublin, Ireland; 6 Centre for Advanced Medical Imaging (CAMI), St. James’s Hospital, Dublin, Ireland; 7 Neurology Department, St. James’s Hospital, Dublin, Ireland; 8 Neurology Department, Cork University Hospital, Cork, Ireland; 9 Division of Neurology, Beaumont Hospital, Dublin, Ireland; Institute of Psychology, Chinese Academy of Sciences, China

## Abstract

**Objectives:**

We aimed to 1) determine if subcortical volume deficits are common to mesial temporal lobe epilepsy (MTLE) patients and their unaffected siblings 2) assess the suitability of subcortical volumetric traits as endophenotypes for MTLE.

**Methods:**

MRI-based volume measurements of the hippocampus, amygdala, thalamus, caudate, putamen and pallidium were generated using an automated brain reconstruction method (FreeSurfer) for 101 unrelated ‘sporadic’ MTLE patients [70 with hippocampal sclerosis (MTLE+HS), 31 with MRI-negative TLE], 83 unaffected full siblings of patients and 86 healthy control subjects. Changes in the volume of subcortical structures in patients and their unaffected siblings were determined by comparison with healthy controls. Narrow sense heritability was estimated ipsilateral and contralateral to the side of seizure activity.

**Results:**

MTLE+HS patients displayed significant volume deficits across the hippocampus, amygdala and thalamus ipsilaterally. In addition, volume loss was detected in the putamen bilaterally. These volume deficits were not present in the unaffected siblings of MTLE+HS patients. Ipsilaterally, the heritability estimates were dramatically reduced for the volume of the hippocampus, thalamus and putamen but remained in the expected range for the amygdala. MRI-negative TLE patients and their unaffected siblings showed no significant volume changes across the same structures and heritability estimates were comparable with calculations from a healthy population.

**Conclusions:**

The findings indicate that volume deficits for many subcortical structures in ‘sporadic’ MTLE+HS are not heritable and likely related to acquired factors. Therefore, they do not represent suitable endophenotypes for MTLE+HS. The findings also support the view that, at a neuroanatomical level, MTLE+HS and MRI-negative TLE represent two distinct forms of MTLE.

## Introduction

Temporal lobe epilepsy (TLE), the most prevalent form of partial epilepsy in adults, has long been considered an acquired condition. However, epidemiological evidence has indicated a significant genetic predisposition [1], [2]. Several forms of familial TLE have been recognized and often present with patterns of autosomal dominant inheritance and incomplete penetrance [3]. In non-familial (sporadic) forms of TLE, the underlying genetic architecture appears complex and a number of susceptibility genes are believed to interact with several environmental factors to produce the disease phenotype [4].

Based on seizure semiology, TLE can be classified into mesial and lateral subtypes. The pathologic hallmark of the more common mesial TLE (MTLE) is hippocampal sclerosis (HS), which is identified in approximately 65–70% of patients [5]. HS is characterized histologically by cellular loss and synaptic reorganization in particular hippocampal sub-fields and often can be detected using magnetic resonance imaging (MRI) through the identification of hippocampal atrophy and MR signal abnormalities [6]. In the remaining 25–30% of MTLE patients, despite having similar seizure semiology to MTLE+HS, no evidence of hippocampal abnormalities can be identified by MRI. This group of patients is often referred to as MRI-negative TLE.

Quantitative MRI (QMRI) studies of MTLE+HS patients have detected a variety of extra-hippocampal neuroanatomical abnormalities, predominantly ipsilateral to the side of seizure focus, including volume deficits in other subcortical structures (e.g., the amygdala, thalamus, and basal ganglia) [7], [8], mesial temporal regions [7], the cerebellum [9], and several neocortical regions [10]. In contrast, more subtle structural changes have been described in MRI-negative TLE [11]–[13]. Although tissue damage and atrophy is at the root of volumetric deficits, the underlying cause of these neuroanatomical changes remains poorly understood. It remains unknown whether these regional volumetric traits are present prior to the onset of epilepsy or they represent sequelae of the illness. It has been proposed that extra-hippocampal brain atrophy might be induced by recurrent seizure activity spreading from the hippocampus and its adjacent brain regions that participate in seizure generation (e.g. the amygdala) [14]. Structural changes within the hippocampus and neighbouring regions may, however, precede the onset of epilepsy and thus represent a risk factor for MTLE. Kobayashi and colleagues (2002) investigated hippocampal structure in first-degree relatives of patients with familial MTLE+HS and found subtle hippocampal abnormalities in up to 34% of the unaffected relatives of patients, including hippocampal atrophy [15]. Such findings indicate that structural abnormalities within the hippocampus may be present prior to onset of familial MTLE+HS and are determined by a strong genetic predisposition [15].

In sporadic forms of MTLE, if subcortical volume deficits (including hippocampal atrophy) are under the direct control of genetic variation, then volume changes can be expected in unaffected first-degree relatives of patients and the heritability of the impacted structures should be high. Accordingly, subcortical volumetric traits may represent endophenotypes reflecting particular genetic risk factors highly relevant to the condition. In the present study, we examined the changes and heritability of the volume of subcortical structures in 101 unrelated patients with ‘sporadic’ MTLE and 83 unaffected full siblings of patients. We aimed to:

1. Determine if volumetric changes in subcortical structures are common to both ‘sporadic’ MTLE cases and their unaffected siblings.
2. Assess the suitability of volume measures of subcortical structures as endophenotypes for MTLE, the identification of which could aid in the mapping of genetic factors influencing the development of this condition.

## Methods

### Ethics

The following research ethics committees independently approved this study:

1. Beaumont Hospital Ethics (Medical Research) Committee.
2. St. James’s Hospital/AMNCH Research Ethics Committee.
3. Clinical Research Ethics Committee of the Cork Teaching Hospitals.

Written informed consent was provided by all study participants.

### Participants

#### Patients

One hundred and one unrelated patients with mesial temporal lobe epilepsy (MTLE) were recruited from three tertiary epilepsy centres in Ireland: Beaumont Hospital, St. James’s Hospital, and Cork University Hospital. Patients underwent a comprehensive evaluation that confirmed the clinical features of MTLE and the side of seizure activity based on the International League Against Epilepsy (ILAE) guidelines [16]. This evaluation included a combination of detailed history of seizure semiology, ictal/inter-ictal electroencephalography (EEG) and video-telemetry recordings. Based on standard qualitative examination of routine MR images by a neuroradiologist, 70 patients (male/female: 30/40) had evidence of hippocampal sclerosis (MTLE+HS: left/right 35/35) and 31 patients (male/female: 12/19) had normal MRI (MRI-negative TLE). Patients with evidence of any lesion other than hippocampal sclerosis were excluded. The sample of MTLE+HS patients included in this study overlapped (n = 67) with that used in previous reports [17], [18]. Mean age (± standard deviation, SD) was 36.6±11.4 years in the MTLE+HS patients and 37.2 years ±9.6 in the MRI-negative TLE patients. Patients with a family history of epilepsy or febrile seizures in first-degree relatives were excluded. A detailed description of the study participants is presented in Table 1.

**Table 1:** Participants demographics.

	Group 1 (1.5T GE Signa scanner)			Group 2 (3.0T Philips Achieva scanner)		
	HealthyControls	MTLE+HS	Unaffected siblingsof MTLE+HS	Healthycontrols	MRI-negativeTLE patients	Unaffected siblings ofMRI-negative TLE
**Number**	58	70	50	28	31	33
**Age: mean (SD)**	31.5 (9.0)	36.6 (11.4)	36.8 (10.3)	34.7 (8.8)	37.2 (9.6)	37.1 (9.8)
**Gender: number (%)**						
**Male**	27 (46.6%)	30 (42.9%)	20 (40%)	12 (42.9%)	12 (38.7%)	12 (36.4%)
**Female**	31 (53.4%)	40 (57.1%)	30 (60%)	16 (57.1%)	19 (61.3%)	21 (63.6%)
**Side of seizure activity**						
**Left**	–	35	27*	–	12	12*
**Right**	–	35	23*	–	12	12*
**Bilateral**	–	0	0	–	3	2*
**Undetermined**	–	0	0	–	5	7*
**Age at onset: mean (SD)**	–	14.2 (11.3)	–	–	17.8 (10.9)	–
**Epilepsy duration: mean (SD)**	–	22.4 (14.4)	–	–	19.2 (12.3)	–
**IPIs: number (%)**						
**Febrile Seizures**	0	33 (47.1%)	0	0	6 (19.4%)	0
**Intracranial Infection(s)**	0	4 (5.7%)	0	0	1 (3.2%)	0
**Status epilepticus**	0	9 (12.8%)	0	0	2 (6.4%)	0
**Underwent** **Surgery: number (%)**	–	32 (45.7%)	–	–	1 (3.2%)	–

SD: standard deviation; IPIs: initial precipitating insults;

* indicates the number of unaffected siblings based on the side of seizure activity in patients.

#### Unaffected siblings

In total, 83 unrelated, asymptomatic (for seizures), same-gender full siblings of MTLE patients were also recruited. Of those, 50 were siblings of MTLE+HS patients (male/female: 20/30) and 33 were siblings of MRI-negative TLE patients (male/female: 12/21). The mean age difference between patients and their unaffected siblings was 5.1 years (range: 1–13). Siblings with known neurological or psychiatric illness were excluded. Further, those with a positive history of childhood febrile seizures, intracranial infection or significant head trauma were also excluded. See Table 1 for additional details.

#### Controls

Our control population consisted of 86 healthy individuals with no known neurological or psychiatric illness (male/female: 40/46). Controls with a history of childhood febrile seizures, intracranial infection or significant head trauma were excluded. Control subjects with a family history of epilepsy or febrile seizures were also excluded.

### MR Image Acquisition

Participants were divided into two groups for the purpose of MR image acquisition.

#### Group 1

MTLE+HS patients (n = 70), their unaffected siblings (n = 50), and healthy control subjects (n = 58) were scanned using a 1.5 T MRI scanner (Signa, GE, Milwaukee, WI, USA) at Beaumont Hospital. A three-dimensional (3D) T_1_-weighted spoiled gradient recalled sequence (TR/TE = 10.1/4.2 ms, ms, TI = 450 ms, flip angle = 20°, field of view = 24×24 cm^2^, matrix = 256×256) with 124 sagittal slices (slice thickness = 1.5 mm) was used to acquire the images.

#### Group 2

MRI-negative TLE patients (n = 31), their unaffected siblings (n = 33), and healthy control subjects (n = 28) were scanned using a 3.0 T MRI scanner (Achieva, Philips Medical Systems, The Netherlands) at the Centre for Advanced Medical Imaging, St. James’s Hospital. A three-dimensional (3D) T_1_-weighted turbo field echo sequence (TR/TE = 8.5/3.9 ms, flip angle = 8° turbo factor n = 240, field of view = 25.6×25.6 cm^2^) with 160 slices and an isotropic spatial resolution of 1.0 mm^3^ was used to acquire the images.

### MR Image Processing

MR images were processed using FreeSurfer, a fully automated image analysis software (version 4.50). The FreeSurfer process has been described in detail previously [19]–[22]. It has undergone extensive investigations to assess its accuracy, validity and applicability [23]. We applied FreeSurfer to segment and produce volume measurements of the subcortical structures and total intracranial volume (ICV) as described in Alhusaini et al [17]. Careful visual quality checks of all segmentation and cortical reconstructions were performed as recommended by FreeSurfer software guidelines.

### Data Analysis and Statistics

#### Analysis of MRI-based subcortical volume measurements

For each participant, MRI-based volume measurements were generated for the following subcortical structures in each hemisphere: the hippocampus, thalamus, amygdala, putamen, pallidum, and caudate. Data from group 1 and 2 (that is, participants scanned using the 1.5T GE Signa and the 3T Philips Achieva systems, respectively) were analysed independently and accordingly no formal comparisons were made across the two MRI-platforms. Multivariate ANCOVA’s (covariates: estimated ICV, age, and gender) were employed to compare volume measurements of the subcortical structures between MTLE patients and their unaffected siblings to the healthy controls. Unpaired t-tests were used to test for significant mean differences.

MTLE+HS patients were divided into left (n = 35) and right (n = 35) MTLE+HS based on the side of seizure activity/HS and were compared separately to the healthy controls scanned on the 1.5T scanner (n = 58). Similarly, their unaffected siblings were divided into siblings of left MTLE+HS patients (n = 27) and siblings of right MTLE+HS patients (n = 23) and each group was compared to the same healthy controls.

MRI-negative TLE patients with left (n = 12) and right (n = 12) seizure activity focus were compared separately to the healthy control group (n = 28) scanned on the 3T scanner. MRI-negative TLE patients with bilateral (n = 2) or undetermined (n = 5) side of seizure activity were excluded from the analysis. Siblings of left and (n = 12) right (n = 12) MRI-negative TLE patients were also treated as separate groups and were compared independently to the same healthy controls (n = 28).

Correction for multiple comparisons were made using a false discovery rate (FDR), setting the level of significance at *p = *0.05 [24].

#### Heritability calculations

The heritability (i.e., the fraction of phenotype variability that can be attributed to genetic variation*)* of the volume of each subcortical structure was estimated in MTLE patients and their unaffected siblings using the maximum-likelihood variance components model implemented in the Sequential Oligogenic Linkage Analysis Routines (SOLAR) version 2.1.4 software package [25]. The software was applied to set up a model that used the pedigree covariance matrix which included information on MTLE patients and their unaffected siblings. In this model, the phenotypic variance, σ_p_^2^, for a quantitative trait was decomposed into a genetic component, σ_g_^2^, and a residual component that includes all effects not accounted for by the genetic component, σ_e_^2^, while accounting for the population and pedigree structure using the following equation [25].
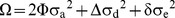
where Ω is the covariance between two relatives, Φ is the coefficient of relationship between the two relatives, σ_a_^2^ is the additive genetic variance, Δ is the probability that the two relatives share both alleles identical by decent, σ_d_^2^ is the dominant genetic variance, δ is the identity matrix, and σ_e_^2^ is the variance accounted by non-genetic variation, including random environmental effects and experimental errors. For any pair of relatives, the value for Φ and Δ can be estimated. For example, for full sibling pairs, the values are ½ and ¼ respectively.

The term heritability is commonly associated with the narrow sense heritability (*h^2^*) which is estimated as the ratio of σ_g_^2^to σ_p_^2^ where the genetic component (σ_g_^2^ *)* of the phenotypic variance is defined by an additive genetic component (σ_a_^2^).

In order to determine the influence of the disease, the narrow-sense heritability (*h^2^*) of the volume of each subcortical structure was estimated ipsilateral and contralateral to the side of seizure activity. For this analysis, regional volumetric measurements for the patients and their discordant siblings were re-assembled into ipsilateral and contralateral measurements according to the side of seizure focus in patients. A separate univariate model was set up to calculate the heritability for each trait (i.e. the volume of each subcortical structure) with ICV, gender, and age included as covariates. Heritability estimates were compared to those reported for healthy middle-aged male twins (n = 474) by Kremen et al [26].

## Results

### Comparison of MTLE+HS Patients and their Unaffected Siblings to the Healthy Controls

**Figure 1:**
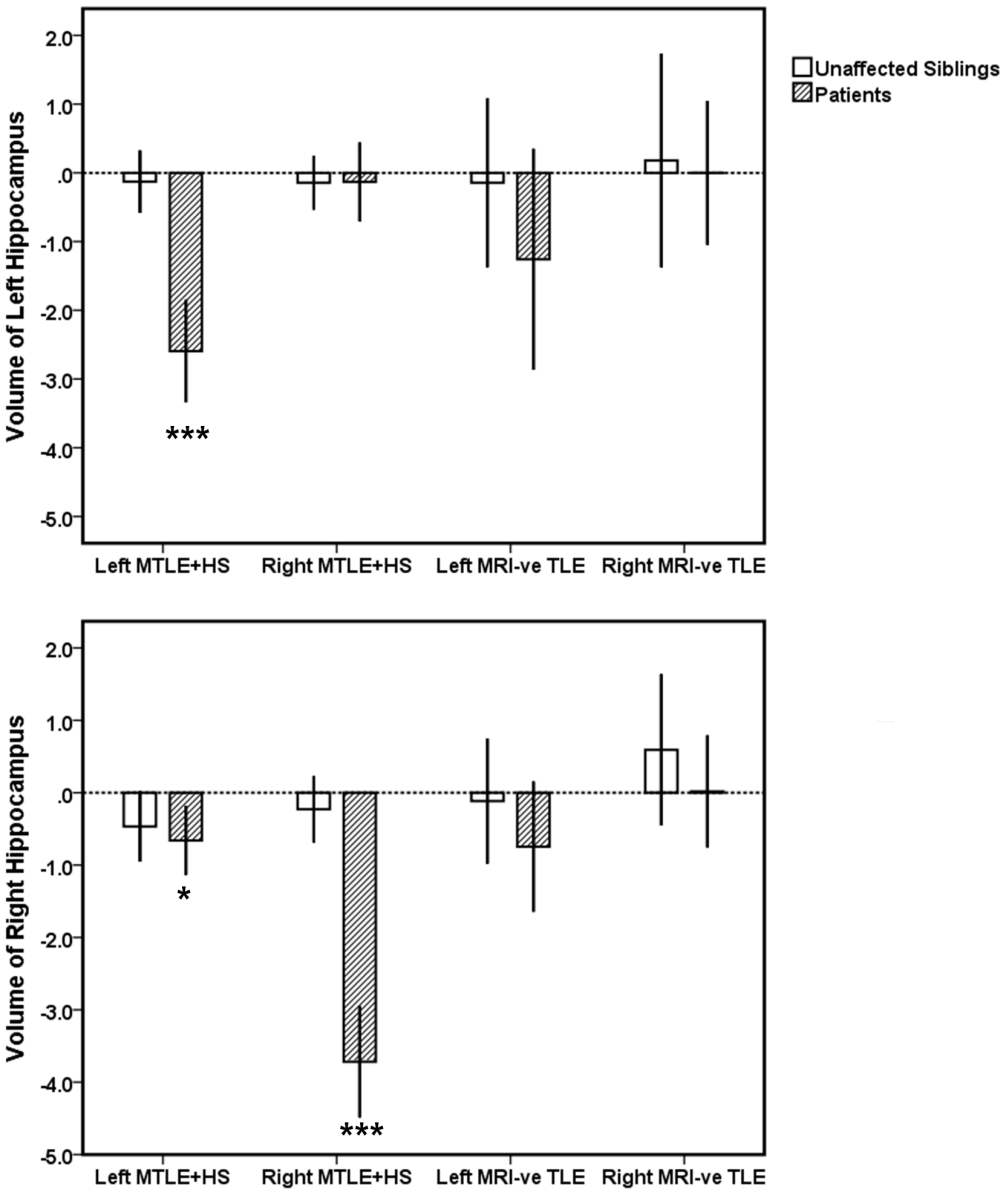
The volume of left (top panel) and right (bottom panel) hippocampus in MTLE patients and their unaffected siblings relative to the healthy controls. Volume measurements are reported in z-scores which were derived from the mean of the controls data. Error bands represent 95% confidence intervals (CI). *** Mean is significantly different from the controls at *p*<0.001; *p<0.05 (corrected for multiple comparisons).

**Figure 2:**
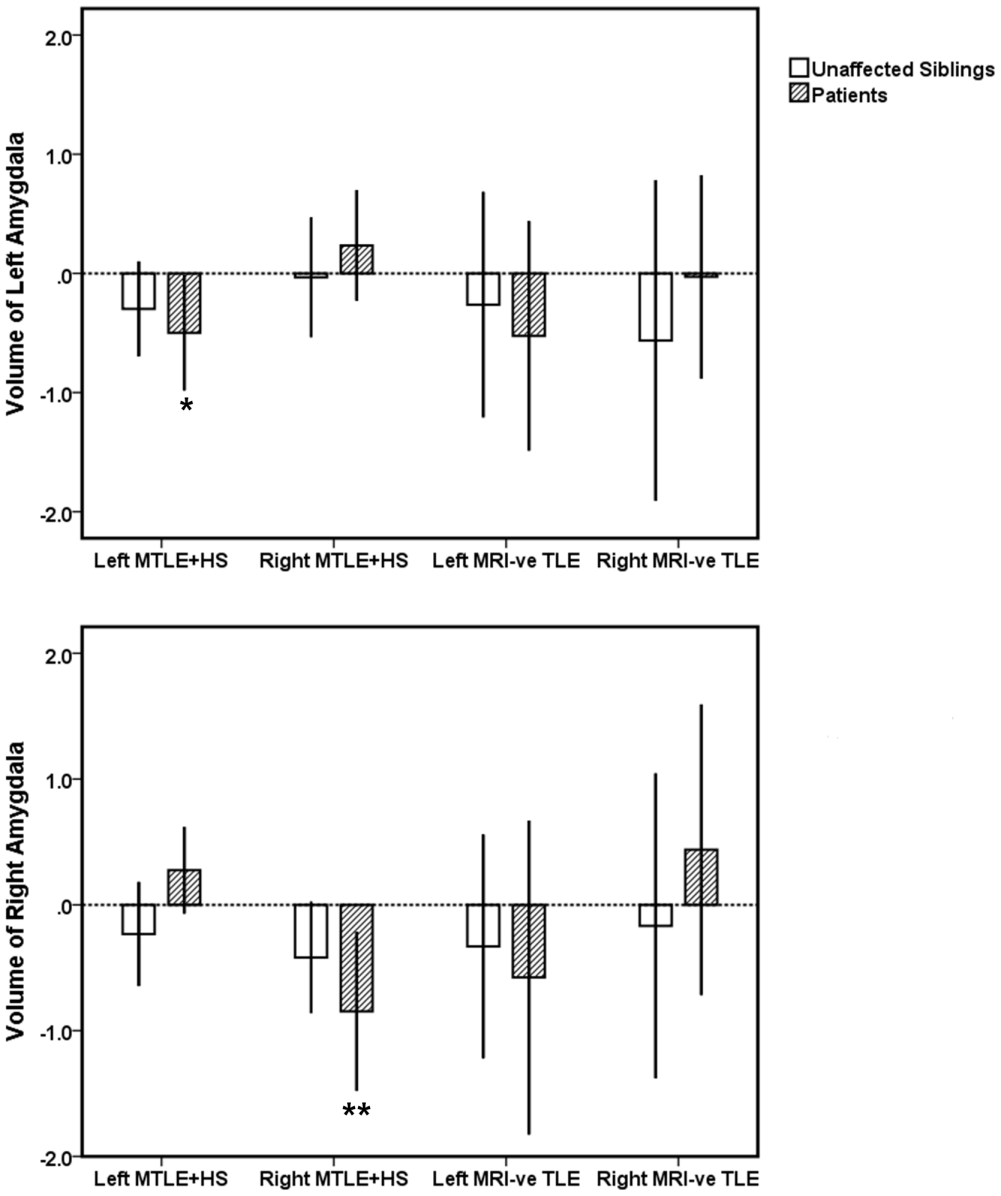
The volume of left (top panel) and right (bottom panel) of amygdala in MTLE patients and their unaffected siblings relative to the healthy controls. Volume measurements are reported in z-scores which were derived from the mean of the controls data. Error bands represent 95% confidence intervals (CI). ** Mean is significantly different from the controls at *p*<0.01; *p<0.05 (corrected for multiple comparisons).

**Figure 3:**
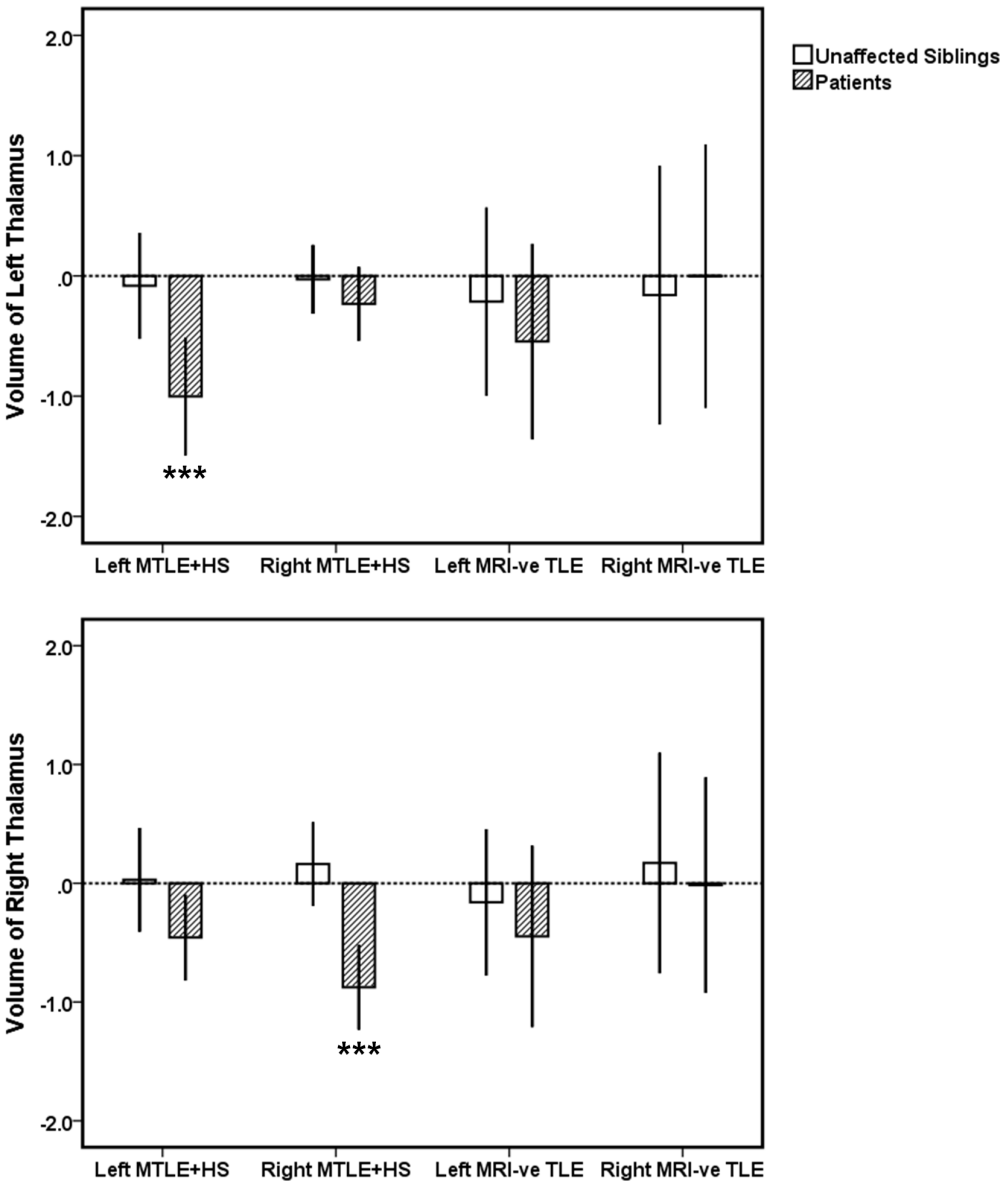
The volume of left (top panel) and right (bottom panel) thalamus in MTLE patients and their unaffected siblings relative to the healthy controls. Volume measurements are reported in z-scores which were derived from the mean of the controls data. Error bands represent 95% confidence intervals (CI). *** Mean is significantly different from the controls at *p*<0.001; (corrected for multiple comparisons).

Comparing patients to controls, left and right MTLE+HS patient groups displayed significant volume deficits in ipsilateral hippocampus (left and right MTLE+HS: *p*<0.0001), amygdala (left MTLE+HS: *p* = 0.02; right MTLE+HS: *p* = 0.007), and thalamus (left and right MTLE+HS: *p*<0.0001) (see Figures 1, 2, 3). Additionally, significant bilateral volume deficits were observed in the putamen in both left (ipsilateral: *p* = 0.003, contralateral *p* = 0.011) and right MTLE+HS patient groups (ipsilateral *p* = 0.007; contralaeral: *p* = 0.017). Patients with left MTLE+HS also displayed volume reduction in contralateral hippocampus (*p* = 0.012). No significant volume differences across the same structures were detected between the unaffected siblings of MTLE+HS patients and the healthy controls. However, a non-significant trend of volume reduction in ipsilateral amygdala was observed (siblings of left MTLE+HS patients: *p* = 0.09; siblings of right MTLE+HS patients: *p* = 0.08); see Figures 1–3 and Figures S1, S2, S3. All reported significant *p*-values survived correction for multiple comparisons (FDR correction threshold *p* = 0.05).

### Comparison of MRI-negative TLE Patients and their Unaffected Siblings to the Healthy Controls

No significant volume change was observed in MRI-negative TLE patients when compared to healthy controls. Similarly, their unaffected siblings showed no significant volume changes when compared to the healthy controls. Results are shown in Figures 1, 2, 3 and Figures S1, S2, S3.

### Heritability of the Volume of Subcortical Structures

The results of the heritability estimates for the volume of subcortical structures calculated in MTLE patients and their unaffected siblings ipsilateral and contralateral to the side of seizure focus are shown in Table 2. In MTLE+HS, the heritability of the volumes of all subcortical structures ipsilateral to the side of HS appeared reduced when compared to those on the contralateral side, with the exception of the amygdala. The volume of ipsilateral hippocampus showed no evidence of heritability (*h^2^* = 0) and that of ipsilateral thalamus showed very small evidence of heritability (*h^2^* = 0.04). Further, reduced heritability was observed for the volume of ipsilateral caudate (*h^2^* = 0.28), putamen (*h^2^* = 0.35) and pallidum (*h^2^* = 0.40). In contrast, high heritability was noted for the volume of ipsilateral amygdala (*h^2^* = 0.81). The heritability estimates for the volume of contralateral subcortical structures were higher than those estimated ipsilaterally (ranged from 0.43–0.83) and were relatively comparable to those reported in healthy individuals [26].

**Table 2:** Heritability estimates for the volume of subcortical structures in MTLE+HS and MRI-negative TLE.

Group	MTLE+HS				MRI-negative TLE				Reported for healthy middle-aged male twins (n = 474) [26]			
Hemisphere	Ipsilateral		Contralateral		Ipsilateral		Contralateral		Left		Right	
Subcorticalstructure	*h^2^*	SE	*h^2^*	SE	*h^2^*	SE	*h^2^*	SE	*h^2^*	95% CI	*h^2^*	95% CI
Hippocampus	0	0.32	0.43	0.22	0.83	0.23	0.85	0.22	0.63	(0.36–2)	0.64	(0.47–0.74)
Amygdala	0.81	0.21	0.83	0.19	0.42	0.31	0.53	0.29	0.63	(0.28–0.72)	0.66	(0.33–0.74)
Thalamus	0.04	0.22	0.42	0.21	0.73	0.16	0.77	0.19	0.68	(0.35–0.77)	0.60	(0.30–0.81)
Caudate	0.28	0.23	0.61	0.17	0.74	0.27	0.79	0.20	0.79	(0.54–0.91)	0.70	(0.43–0.86)
Putamen	0.35	0.19	0.55	0.20	0.69	0.28	0.76	0.25	0.85	(0.56–0.90)	0.84	(0.63–0.88)
Pallidum	0.40	0.22	0.80	0.21	0.68	0.19	0.84	0.19	0.66	(0.33–0.78)	0.75	(0.44–0.81)

The heritability was calculated ipislateral and contralateral to side of seizure focus. Estimated ICV, gender, and age were included as covariates. h^2^: narrow-sense heritability, SE: standard error, CI: confidence interval.

In MRI-negative TLE, the heritability estimates for the volume of all ipsilateral and contralateral subcortical structures ranged from 0.42–0.85 and were comparable to those reported by Kremen et al [26].

## Discussion

In the present study, the volume and heritability of brain subcortical structures were examined in a large sample of unrelated patients with ‘sporadic’ MTLE and their unaffected siblings. The results indicate that subcortical volume deficits are significant in MTLE+HS but apparently absent from MRI-negative TLE patients. Subcortical volume deficits were not present in the unaffected siblings of patients. These findings suggest that, in sporadic MTLE+HS, most of the significant volume deficits in subcortical structures are largely determined by non-genetic factors.

Previous QMRI-based studies that investigated subcortical structures in patients with MTLE+HS have consistently identified volume loss in ipsilateral hippocampus, amygdala and thalamus [7], [8], [10]. The significance of such subcortical atrophy remains poorly understood as does whether these abnormalities represent sequelae of the disease or are present prior to the onset of epilepsy. QMRI-based neuroanatomical traits have been proposed as potential endophenotypes for epilepsy [27], [28]. To qualify as endophenotypes, these traits must (1) associate with the illness (2) be independent of disease state and (3) be heritable. In a previous report studying familial MTLE+HS patients, hippocampal atrophy was identified in 34% of the unaffected first-degree relatives, suggesting that hippocampal abnormality in familial MTLE+HS is heavily influenced by genetic factors [15]. In the present study, with the exception of a non-significant trend in the amygdala, MTLE+HS-related subcortical volume deficits were not observed in the unaffected full siblings of ‘sporadic’ MTLE+HS patients. These findings indicate that, for sporadic MTLE+HS, while genetic factors may still play a role in determining volume reduction in ipsilateral amygdala, volume deficits in ipsilateral hippocampus, thalamus, and putamen are state dependent, not heritable and likely related to non-genetic factor(s). Studying three monozygotic twins discordant for MTLE+HS, Jackson and colleagues found no evidence of hippocampal atrophy in the unaffected twins [29]. Although the study sample was small, lack of hippocampal abnormalities in the unaffected twins argues against a strictly genetic basis for hippocampal atrophy [29].

Several studies have reported significant correlations between duration of epilepsy and MTLE+HS-related volume deficits in ipislateral hippocampus, amygdala and thalamus, indicating progressive volume loss [17], [30], [31]. In addition, an association between hippocampal sclerosis and initial precipitating insults (IPIs), such as febrile seizures, head trauma and CNS infection has long been considered [32]. Evidence of hippocampal damage secondary to recurrent seizure activity has also been suggested by some longitudinal MRI studies [33], [34]. Cumulative damage caused by several environmental and disease-related factors, including IPIs and epilepsy chronicity, may therefore explain the underlying processes responsible for the progressive volume loss in the hippocampus and the other subcortical structures in ‘sporadic’ MTLE+HS. Hence, these subcortical volumetric traits are unlikely to be present prior to epilepsy onset and thus they do not represent suitable endophenotypes for MTLE+HS. One possible exception is the amygdala. Given the high heritability values observed for the volume of the amygdala, this structure may qualify as a potential MTLE+HS-related endophenotype, although further work is required to confirm this. It should be noted that the amygdala is generally a difficult structure to segment accurately using fully automated methods. Although FreeSurfer segmentation of the amygdala has previously been found to correlate highly with manual tracing, amygdalar anterior and posterior surfaces are usually inflated [35]. The increased variability in volume measures is likely to influence heritability estimates and thus our heritability scores are possibly conservative.

In the current study, MRI-negative TLE patients and their unaffected siblings displayed no evidence of volume deficits across any of the subcortical structures. The lateralization effect of the seizure activity, which was very apparent in the MTLE+HS group, was absent in the MRI-negative TLE patients group. This was reflected by the heritability estimates where no difference was noted between the ipsilateral and contralateral subcortical structures. These observations may reflect the subtlety of subcortical structural abnormalities in MRI-negative TLE or indicate the involvement of distinct epileptic networks. Subtle atrophy affecting particular hippocampal subfields was previously reported in a small number of MRI-negative TLE patients in a pattern that was different from that usually seen in MTLE+HS patients [12]. In contrast to a unilateral CA1 subfield atrophy in MTLE+HS, Mueller and colleagues found 17% of MRI-negative TLE patients to display non-lateralizing hippocampal subfield volume deficits sparing CA1 [12]. Clinically, MRI-negative TLE is very heterogeneous and despite displaying similar seizure semiology to MTLE+HS, the epileptiform discharges in patients are often diffuse and difficult to lateralize [36], [37]. This was evident in our sample. Seven out of 31 of MRI-negative patients (22.6%) showed no lateralizing seizure semiology features or epileptiform EEG abnormalities. Previous positron emission tomography (PET) studies of MRI-negative TLE patients reported lateralizing metabolic abnormalities involving lateral temporal lobe regions [38]. These findings suggest the involvement of different epileptic networks in MTLE+HS and MRI-negative TLE.

In the present study, we focused on volumetric measures of subcortical structures. Future work is required to assess MTLE-related cortical grey matter alterations, including neocortical thinning [39], surface area changes [18], and folding alterations [40], which may represent suitable endophenotypes, especially in MRI-negative TLE.

## Supporting Information

Figure S1 The volume of left (top panel) and right putamen (bottom panel) in MTLE patients and their unaffected siblings relative to the healthy controls. Volume measurements are reported in z-scores which were derived from the mean of the controls data. Error bands represent 95% confidence intervals (CI). **Mean is significantly different from the controls at *p*<0.01; *p<0.05 (corrected for multiple comparisons).
(TIF)

Figure S2 The volume of left (top panel) and right (bottom panel) of caudate in MTLE patients and their unaffected siblings relative to the healthy controls. Volume measurements are reported in z-scores which were derived from the mean of the controls data. Error bands represent 95% confidence intervals (CI).
(TIF)

Figure S3 The volume of left (top panel) and right (bottom panel) pallidum in MTLE patients and their unaffected siblings relative to the healthy controls. Volume measurements are reported in z-scores which were derived from the mean of the controls data. Error bands represent 95% confidence intervals (CI).
(TIF)
