# Supplementary figures and images for: Heritability of Subcortical Volumetric Traits in Mesial Temporal Lobe Epilepsy
Source: PLoS One. 2013 Apr 23;8(4):e61880. doi: 10.1371/journal.pone.0061880 (PMC3633933; doi:10.1371/journal.pone.0061880)

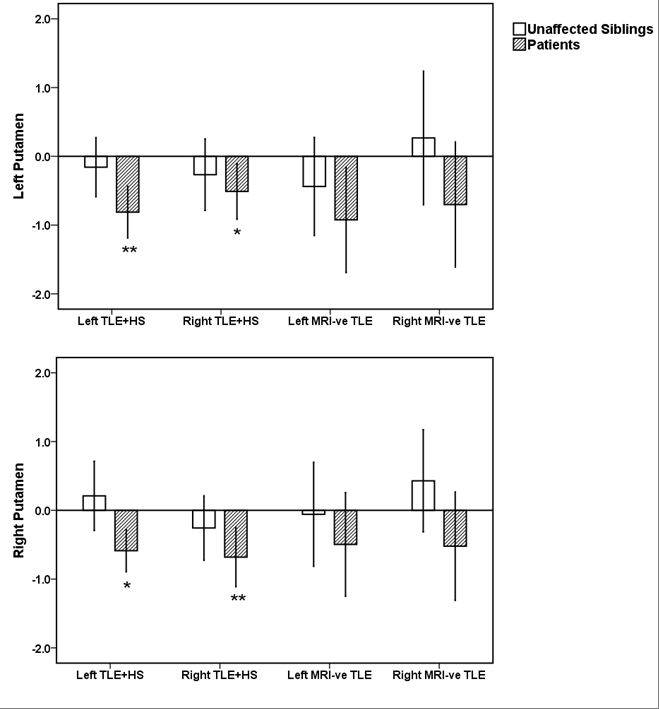

Supplement: Figure S1 — The volume of left (top panel) and right putamen (bottom panel) in MTLE patients and their unaffected siblings relative to the healthy controls. Volume measurements are reported in z-scores which were derived from the mean of the controls data. Error bands represent 95% confidence intervals (CI). **Mean is significantly different from the controls at p<0.01; *p<0.05 (corrected for multiple comparisons). (TIF) [file pone.0061880.s001.tif]
